# Janus Dumbbell Ru–Pt Nanoparticles on Titanium Oxynitride Boost Hydrogen Evolution Across pH

**DOI:** 10.1002/cssc.71203

**Published:** 2026-11-08

**Authors:** Mejrema Nuhanović, Ivan Marić, Marjan Bele, Milena Šetka, Francisco Ruiz‐Zepeda, Naveed Ashraf, Mohammad Isa, Younes Abghoui, Nejc Hodnik, Milutin Smiljanić

**Affiliations:** ^1^ Department of Materials Chemistry National Institute of Chemistry Ljubljana Slovenia; ^2^ University of Nova Gorica Nova Gorica Slovenia; ^3^ Radiation Chemistry and Dosimetry Laboratory Division of Materials Chemistry Ruđer Bošković Institute Zagreb Croatia; ^4^ Institute of Metals and Technology Ljubljana Slovenia; ^5^ Device and Sensor Center Fondazione Bruno Kessler Trento Italy; ^6^ Department of Inorganic Technology University of Chemistry and Technology Prague Prague Czech Republic; ^7^ Science Institute of the University of Iceland Reykjavik Iceland

**Keywords:** hydrogen evolution reaction, metal–support interactions, platinum, ruthenium, titanium oxynitride

## Abstract

While platinum nanocatalysts exhibit outstanding activity for the hydrogen evolution reaction (HER), their high cost and scarcity underscore the need to develop more efficient and sustainable alternatives. This work reports a highly active, stable, and cost‐effective electrocatalytic composite for HER consisting of bimetallic Janus dumbbell Ru─Pt nanoparticles deposited on a titanium oxynitride‐graphene support (Ru─Pt/TiON─C). Ru─Pt/TiON─C nanocomposite with particularly low metal loading of only 1.85 wt.% Pt and 5.5 wt.% Ru outperforms the 20 wt.% Pt/C benchmark catalyst in both acid and alkaline electrolytes. A joint experimental–theoretical approach, combining electrochemical measurements, transmission electron microscopy, X‐ray photoelectron spectroscopy, and density functional theory calculations, reveals that the improved performance and metal utilization stem from the intimate Ru─Pt─TiON interface. These findings point to beneficial support effects arising from TiON, together with Ru‐induced modification of the Pt active sites that promotes more favorable hydrogen adsorption thermodynamics. Such a catalyst structure results in exceptional HER activity of Ru─Pt/TiON─C across the entire pH range.

## Graphical abstract

A low‐loaded Ru─Pt/TiON─C catalyst delivers outstanding HER performance across acidic and alkaline media while markedly improving PGM utilization compared with commercial Pt/C. The enhanced activity arises from the interplay between Janus Ru─Pt active sites and titanium oxynitride support effects, highlighting an efficient strategy for reducing precious‐metal demand in water electrolysis.

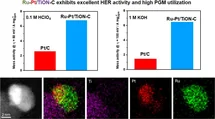

## 1 Introduction

In the last decades, significant attention has been devoted to carbon‐neutral hydrogen production via water electrolysis, where the hydrogen evolution reaction (HER) is the cathodic process complemented by the oxygen evolution reaction at the anode. Platinum is considered a near‐perfect catalyst for HER, as it exhibits facile reaction kinetics at low overpotentials, particularly in acidic electrolytes [1]. The high activity of Pt stems from its near‐optimal hydrogen adsorption strength, expressed by a Gibbs energy for dissociative adsorption of hydrogen (Δ*G*_H*_) value close to zero, which ensures an optimal balance between hydrogen adsorption and desorption, placing Pt near the apex of the HER activity volcano plot [2, 3]. In alkaline media, HER on Pt is notably slower than in acid due to an additional kinetic penalty introduced by sluggish water activation [4]. Various alternative materials have been explored, aiming to match the HER performance of Pt, including MoS_2_ [5, 6], transition‐metal phosphides [7, 8], carbides [9, 10], and nitrides [11]. Despite being much more cost‐effective, these catalysts still fail to match the intrinsic performance of Pt. Therefore, Pt remains a reference catalyst for both acid and alkaline HER and finds practical applications in both proton exchange membrane and anion exchange membrane water electrolyzers [12, 13, 14]. Since Pt is an expensive and scarce resource, further optimization of HER catalysts should primarily focus on improving Pt utilization while maintaining its high performance. Various strategies can be explored to achieve this, including advanced nanostructuring [15], strain engineering [16], single‐atom‐based catalysts [17], multimetallic nanostructures [18, 19], high‐entropy alloys [20], and support engineering [15, 21].

Our group recently reported that metal–support interaction (MSI) is an effective means to enhance the reactivity of different active sites for HER using titanium oxynitride (TiON) as a support material [21, 22, 23]. In all of these works, carbon support templates were combined with TiON, aiming to improve its dispersion and increase surface area. The intrinsic HER activity of TiON‐supported Pt nanoparticles was improved compared to the carbon‐supported Pt/C reference by favorable tuning of the Δ*G*_H*_ parameter, whereas the stronger anchoring of the Pt nanoparticles to the TiON, with respect to carbon, resulted in enhanced durability [21]. The mentioned Pt/TiON catalyst was loaded with a similar amount of Pt as the commercial Pt/C benchmarks (~20 wt.%), leaving room for further improvement in precious metal utilization. In our follow‐up study, Ru nanoparticles with a particularly low loading of only 6 wt.% were supported on TiON [22]. In an acid electrolyte, the Ru/TiON catalyst showed notable activity, reducing the substantial gap between the performance of Ru/C and Pt/C. At the same time, Ru/TiON exhibited excellent alkaline HER properties, surpassing both Ru/C and Pt/C at a significantly lower metal loading. This was ascribed to the particularly strong MSI provided by TiON, improving both hydrogen adsorption and water dissociation properties at Ru‐active sites. Building on this concept, we further demonstrated that MSI can be combined with bimetallic active sites to yield additional performance gains at both low and high pH values, as shown for core–shell Ru@Ir/TiON catalysts [23]. However, given the scarcity and strategic importance of Ir for anodic oxygen evolution, more practical HER catalysts could be built using the same approach by synergizing Ru─Pt active sites with TiON support. Past reports have shown various Ru─Pt nanostructures with exceptional HER activity, which was linked to the electronic and structural optimization [24], improved H adsorption and faster water dissociation [25], and synergistic effects at the Ru─Pt interface [26, 27].

This work presents a highly active catalyst for HER, consisting of low‐loaded Ru─Pt bimetallic nanostructures dispersed over a TiON‐graphene support (further denoted as Ru─Pt/TiON─C). The composite material was characterized using X‐ray diffraction (XRD), high resolution transmission electron microscopy, and X‐ray photoelectron spectroscopy (XPS), while the metal loading was determined using inductively coupled plasma optical emission spectroscopy (ICP‐OES). The Ru─Pt/TiON─C catalyst with only 5.5 wt.% Ru and 1.85 wt.% Pt delivered high HER activity, exceeding that of the commercial Pt/C benchmark in both acid and alkaline electrolytes while showing stable operation. The observed HER performance can be attributed to the carefully engineered active sites, where both the MSI provided by TiON and the presence of Ru on Pt nanostructures are synergized, allowing low‐loaded Pt sites to efficiently drive HER. The proposed concept significantly improved the mass utilization of platinum group metals (PGM) in the Ru─Pt/TiON─C composite, which could be applied in both PEM and AEM water electrolyzers.

## 2 Methodology

### 2.1 Synthesis and Characterization of Ru─Pt/TiON─C Composite

Preparation of the Ru/TiON─C composite is described in detail in our previous work [22]. Briefly, 0.1 g of dried GO was mixed with 1 mL of propanol containing 1 mmol of Ti isopropoxide, followed by hydrolysis with 0.4 mL of ultrapure water and freeze‐drying. Subsequently, 1.5 mL of an acetone solution containing 0.1 mmol RuCl_3_·H_2_O was added to the dried TiO_2_/GO composite and gently ground at 50 °C until solvent evaporation. The resulting material was thermally treated under an NH_3_ flow (100 mL min^−1^), first at 250 °C for 2 h (2 °C min^−1^) and subsequently at 750 °C for 9 h (10 °C min^−1^), followed by cooling to room temperature. The obtained Ru/TiON─C composite contained 6 wt.% Ru, as determined by ICP‐OES. To deposit Pt, a wet‐chemical approach was used. 100 mg of the aforementioned Ru/TiON─C composite was ultrasonicated in 10 mL of ultrapure water for 20 min. Into the mixture, 0.000013 mol of PVP (molecular weight: 40,000) and 0.000039 mol of ascorbic acid were added and heated at 80 °C for 10 min. Lastly, 0.000013 mol of H_2_PtCl_6_·*6*H_2_O (Alfa Aesar) was dissolved in 1–2 mL of ultrapure water and quickly added to the heated mixture with vigorous stirring; the whole suspension was left at 80 °C for 3 h. After the synthesis was complete, the resulting suspension was centrifuged, and the powder was washed with water several times. The obtained powder was freeze‐dried to obtain the final product. For comparison of the HER electrocatalytic properties, a homemade Ru/C catalyst with 10 wt.% Ru was used, while Pt/C benchmark was purchased from Premetek (20 wt.%).

The obtained catalyst was further characterized by XRD, scanning transmission electron microscopy (STEM), and XPS. X‐ray diffractograms were obtained using a D4 Endeavor and a Bruker AXS diffractometer with Cu‐Kα radiation and a Sol‐X energy dispersion detector. For a detailed microstructural study, a Cs probe‐corrected scanning transmission electron microscope (Jeol ARM 200 CF) at 80 kV was used. XPS analyses were performed with a Versa Probe 3 AD device (Phi, Chanhassen, MN, US) using a monochromatic Al Kα X‐ray source at 15 kV and 3.3 mA emission current. The powder samples mounted on double‐sided adhesive tape were analyzed on a 1 × 1 mm^2^ spot with the charge neutralizer active throughout the measurements. High‐resolution (HR) XPS spectra were acquired with a pass energy of 27 eV and a binding energy (BE) step of 0.1 eV, while survey spectra were acquired with a pass energy of 224 eV and a BE step of 0.8 eV. To improve the signal‐to‐noise ratio, each spectrum underwent at least 20 sweeps. The binding energy scale of the XPS spectra was calibrated using the C 1s peak at a BE of 284.4 eV, which corresponds to the partially graphitized carbon support (part) in the samples. Data processing, including fitting, was carried out using CasaXPS software, and the Shirley background subtraction method was consistently applied to all measurements. The Ru 3d spectra were fitted using an asymmetric line shape for the Ru component, with the positions of the Ru 3d5/2 and Ru 3d3/2 peaks constrained by a fixed spin–orbit splitting of 4.17 eV and their area ratio fixed at 3:2 [28, 29]. The Pt 4f spectra were analyzed using a fixed Pt 4f7/2–4f5/2 spin–orbit splitting of 3.33 eV, a fixed doublet area ratio of 4:3, and an asymmetric line shape for the dominant metallic Pt° component [30].

### 2.2 Electrochemical Characterization and HER Investigations

Catalyst inks were prepared by dispersing the catalyst powder in ultrapure water (18.2 MΩ·cm) at a concentration of 1 mg mL^−1^ (unless stated otherwise) using an ice‐cooled ultrasonic bath. Glassy carbon (GC) rotating disk electrodes (RDEs, 0.196 cm^2^, Pine) were hand‐polished with an alumina slurry before catalyst deposition. A 20 µL aliquot of the ink was drop‐cast onto each RDE and dried in a closed desiccator, leading to a total catalyst loading of 102 µg cm^−2^. The resulting films were then coated with 5 µL of Nafion (Sigma, 5% solution in a mixture of lower aliphatic alcohols and water) diluted 1:50 in isopropanol.

Hydrogen evolution reaction activity measurements were conducted in Ar‐saturated acidic or alkaline electrolytes using a thin‐film rotating disk electrode setup. Experiments in alkaline media (1 M KOH, Merck) were performed in a Teflon cell, while a glass cell was used for acidic conditions (0.1 M HClO_4_, Carl Roth). A reversible hydrogen electrode (Hydroflex, Gaskatel) and a glassy carbon rod served as the reference and counter electrodes, respectively. Catalyst films were electrochemically activated by cyclic voltammetry: 50 cycles at 300 mV/s in the potential range of 0.05–1 *V*_RHE_, followed by three additional cycles at 50 mV/s in the same range, to ensure a stable and reproducible response. HER activity was assessed by recording polarization curves at 10 mV/s from 0.1 to −0.2 *V*_RHE_ (alkaline electrolyte) or 0.1 to −0.1 *V*_RHE_ (acidic electrolyte). In acid media, stability was investigated by applying an HER current of 10 mA cm^−2^ over 24 h and by using 2500 potential cycles between 0.1 and −0.1 *V*_RHE_, scan rate 100 mV/s. To evaluate the structural stability of Ru─Pt/TiON─C after HER operation, postmortem characterization was performed using SEM‐EDXS and STEM‐EDXS. Scanning electron microscopy combined with energy‐dispersive X‐ray spectroscopy (SEM‐EDXS) analysis was carried out directly on the catalyst layer deposited on the RDE, which was mounted in the SEM sample holder using a custom‐made connector. For STEM‐EDXS analysis, the catalyst layer was removed from the GC RDE by immersing the electrode in a small volume of isopropanol, followed by brief sonication in an ultrasonic bath, resulting in detachment of the catalyst film. A few microliters of the resulting suspension were then drop‐cast onto a TEM grid and allowed to dry prior to analysis. In alkaline media, only the potentiodynamic degradation test was performed using 5000 potential scans between −0.2 and 0.1 *V*_RHE_ at a scan rate of 100 mV/s and reduced catalyst loading of 51 µg cm^−2^. All polarization curves were collected for at least three independently prepared electrodes of the same catalysts, ensuring reproducibility and reliable comparison.

### 2.3 Density Functional Theory Calculations

Density functional theory (DFT) calculations have been performed using the Vienna ab initio simulation package (VASP) [31] to gain insights into electrocatalytic activity and metal−support interactions. The exchange–correlation interactions were described within the generalized gradient approximation using the Perdew–‍Burke–Ernzerhof (PBE) functional [32]. Grimme’s D3 dispersion correction was included to account for long‐range van der Waals interactions [33]. The cutoff energy used was 450 eV, and a 1 × 1 × 1 Monkhorst‐Pack K‐point mesh was employed. Electronic occupancies were treated using a Fermi–Dirac smearing scheme with an electronic temperature of kBT = 0.1 eV to improve convergence of the Kohn–Sham states. Structural optimization was considered converged when the residual forces on all atoms were below 0.01 eV Å^‒^^1^.

A vacuum of 20 Å was introduced along the z‐direction to avoid periodic interactions, while the x and y boundaries were kept periodic. Two support electrodes, titanium oxynitride (TiON) [21, 23, 34], and graphene (C) 3–5, were modeled in this study, with a 5 × 5 × 1 K‐point, and the TiON supercell was modeled with 225 titanium, 200 nitrogen, and 200 oxygen atoms as the support surface. A 14 × 14 × 1 graphene (C) supercell containing 392 carbon atoms was optimized to model the support surface.

For nanoparticle modeling, bulk hexagonal close‐packed Ru and face‐centered cubic Pt were selected as the parent crystal structures. Monometallic Ru and Pt nanoparticles were independently constructed using the VESTA visualization software by defining polyhedral geometries based on selected crystallographic facets. The nanoparticles were generated by truncating the bulk structures within predefined geometric boundaries, removing atoms outside the selected shape. The particle size was controlled by adjusting the truncation limits, and all generated structures were subsequently fully relaxed using DFT as implemented in the VASP package.

## 3 Results and Discussion

### 3.1 Characterization of Ru─Pt/TiON─C Catalyst

The structural characterization of the Ru─Pt/TiON─C composite using XRD and STEM techniques is given in Figure 1. XRD patterns for Pt/C and Ru/C show characteristic reflections for Pt and Ru, respectively (Figure 1a). TiON‐related reflections can be seen in the XRD patterns of Ru─Pt/TiON─C and TiON─C, whereas the former does not show any distinguishable peaks for Pt and Ru. This can be explained by the presence of very small and finely dispersed nanoparticles with a low loading, similarly like in the case of the Ru/TiON─C composite, which contained Ru particles with an average size of 2.1 nm [22]. To further analyze the sample, STEM imaging has been performed.

**FIGURE 1:**
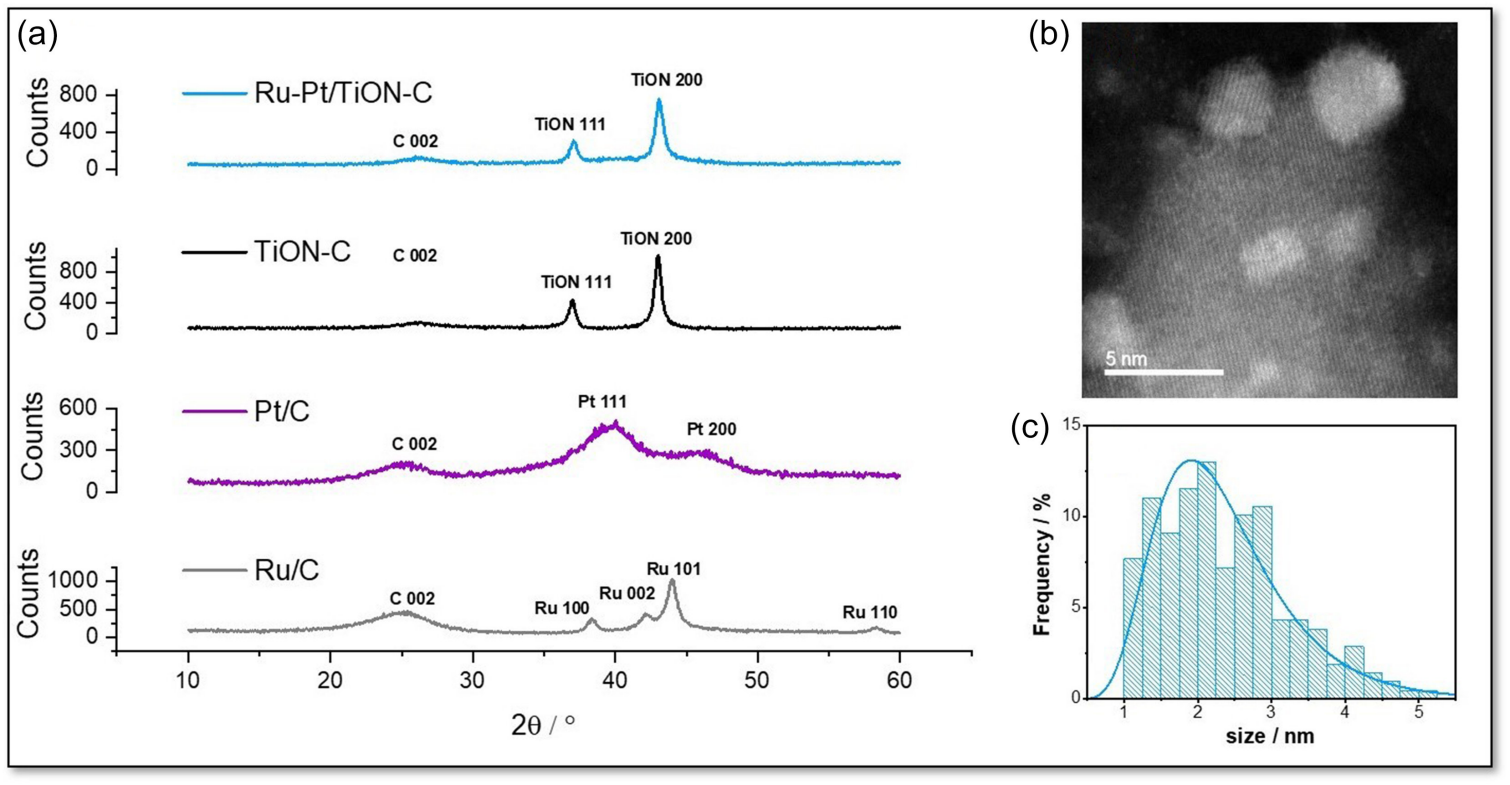
Characterization of Ru─Pt/TiON─C composite: (a) XRD pattern of Ru─Pt/TiON─C compared with TiON─C, Pt/C, and Ru/C; (b) ADF imaging; and (c) corresponding particle size distribution.

High‐resolution annular dark field (ADF) imaging confirmed the presence of small‐sized nanoparticles attached to TiON flakes (Figure 1b). The corresponding particle size distribution shows that most of the particles have a diameter below 3 nm, while the average particle diameter is around 1.95 nm (Figure 1c). The lower magnification image, Figure S1, shows that TiON is finely dispersed over the graphene support, whereas nanoparticles are predominantly anchored to TiON regions, indicating preferential deposition, most likely due to MSI. A similar effect and particle distribution between TiON and the carbon template were already observed in our previous reports [21, 22, 23]. To further analyze the nature of Ru─Pt nanoparticles, a more detailed STEM analysis coupled with EDXS mapping was performed (Figure 2). One can clearly see a TiON flake hosting metallic nanoparticles, as previously suggested. Distribution between C, Ti, O, and N indicates uniform elemental composition of the TiON─C support.

**FIGURE 2:**
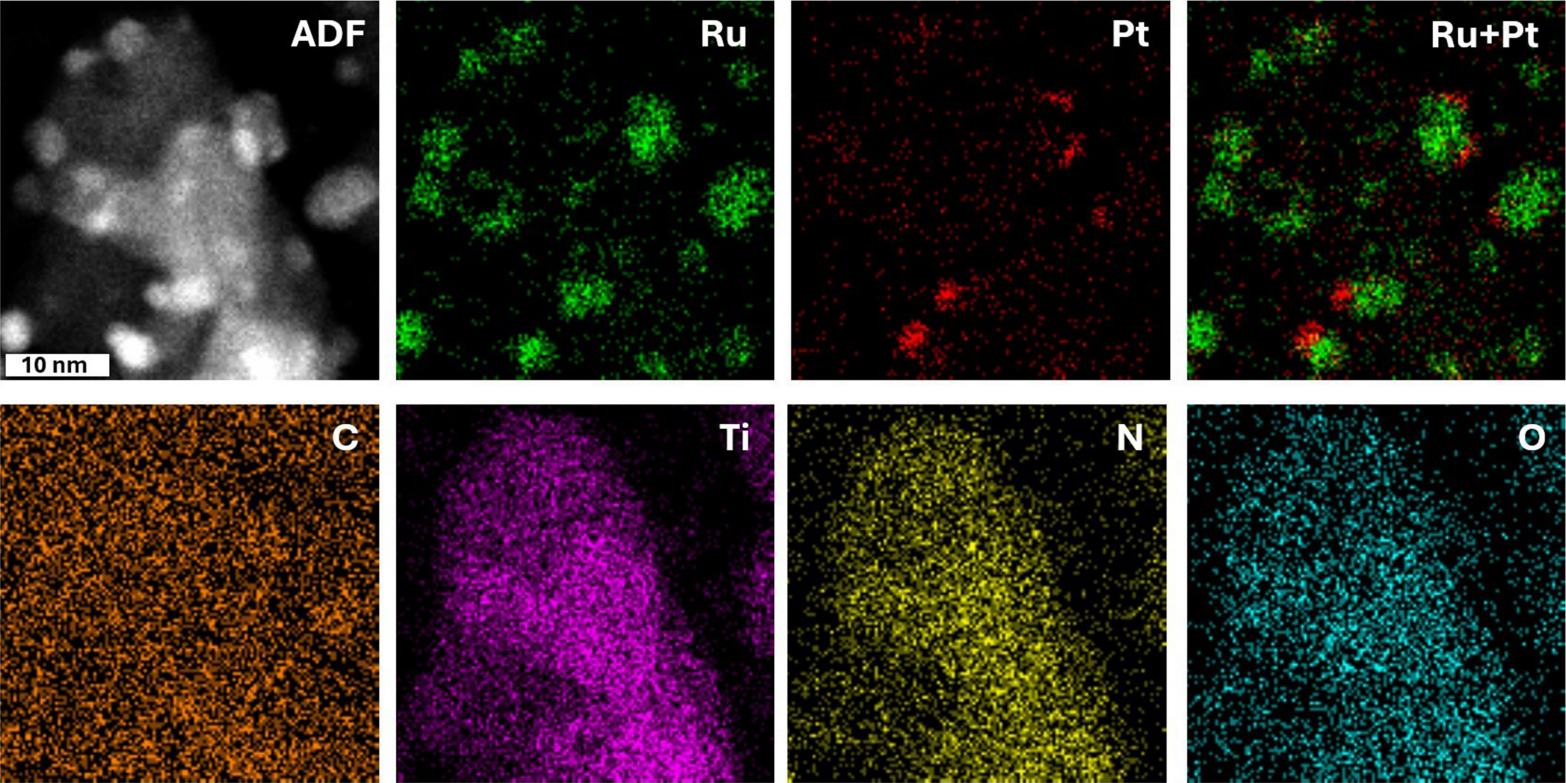
ADF imaging and EDXS mapping of Ru─Pt/TiON─C composite.

Elemental mapping reveals a strong spatial correlation between Ru and Pt signals; however, the two elements are not uniformly distributed within individual particles. The absence of homogeneous overlap between Ru and Pt excludes the formation of an alloy or a well‐defined core–shell architecture. Instead, Pt is preferentially deposited in the vicinity of Ru in the form of an asymmetric structure. This type of morphology, which can be clearly seen in Figure 3, corresponds to particles composed of two distinct but conjoined lobes, giving them a dumbbell shape. They belong to a subclass of Janus particles [35] and could be beneficial for HER electrocatalysis since it offers different but closely connected active sites that may facilitate complementary reaction steps. Further analysis of high‐resolution STEM images (Figure 3a) reveals well‐defined lattice fringes corresponding to both Pt‐ and Ru‐rich domains, while, in addition, lattice fringes associated with the TiON support are observed. The employed sequential synthesis was selected to use the previously developed Ru/TiON─C catalyst as a well‐defined starting platform and to investigate the effect of subsequent Pt incorporation. This synthesis approach resulted in an intimate contact between Ru, Pt, and TiON, an architecture that has been shown to be beneficial for HER electrocatalysis in our previous work [23]. Distinct Pt sites are available to govern hydrogen adsorption/desorption while being influenced by neighboring Ru and the TiON support (Figure 3b). Such an arrangement may provide favorable hydrogen adsorption and water activation properties, consistent with previous reports on Ru‐containing HER catalysts [24, 25, 26].

**FIGURE 3:**
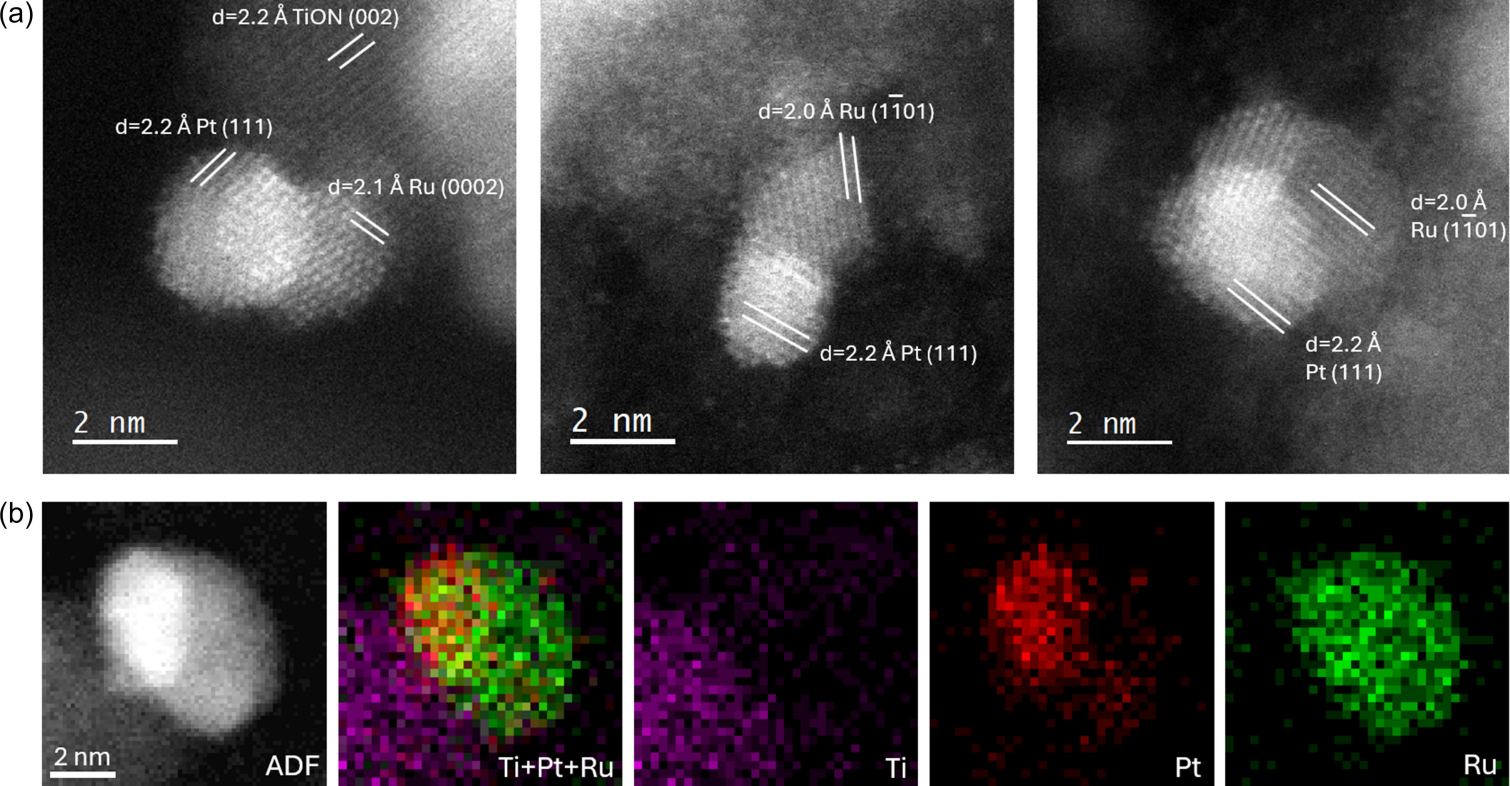
(a) ADF imaging of Ru─Pt nanoparticles with Janus dumbbell‐type characteristics. The morphology suggests a biphasic composition. Pt (Z = 78) and Ru (Z = 44) regions are distinguished by Z‐contrast intensity aside from the overlap. (b) ADF imaging and corresponding EDXS elemental maps for Ti, Pt, and Ru from a nanoparticle exhibiting two distinct Pt‐ and Ru‐rich regions with no observable intermixing.

Insights into the chemical composition and electronic states of the Ru and Pt active sites in the Ru─Pt/TiON─C catalyst were obtained using XPS analysis. Survey spectra for Ru─Pt/TiON─C, together with reference Ru/C and Pt/C benchmarks, are given in Figure S2, confirming the presence of expected elements (Ru, Pt, Ti, C, N, and O) in respective samples. Detailed deconvoluted analyses of high‐resolution spectra of C1, Ru 3d, and Pt 4f are presented in Figure 4 for the respective samples. In the case of Ru, metallic Ru and Ru oxide (RuO_2_) were detected in both Ru/C and Ru─Pt/TiON─C catalysts (Figure 4a,c). The Ru 3d_5/2_ peak is observed at binding energies (BEs) of 280.2 and 279.9 eV for the Ru/C and Ru─Pt/TiON─C samples, respectively. XPS analysis of the Pt 4f region for both Pt/C and Ru─Pt/TiON─C samples (Figure 4b,d) shows contributions from metallic Pt° as well as oxidized Pt species (Pt(OH)_2_ and PtO_2_). The Ru 4s peak overlaps with the Pt 4f region, contributing to the increased intensity of the Pt 4f_5/2_ peak in the Ru─Pt/TiON─C sample. This effect is not observed in Pt/C samples since Ru is not present. Comparison of BEs of Pt 4f_7/2_ peak shows a downshift of 0.4 eV in the case of Ru─Pt/TiON─C (70.9 eV) compared to Pt/C (71.3 eV). These downshifts of Ru0 (0.3 eV) and Pt0 (0.4 eV) peaks suggest a modified electronic environment of both Ru and Pt sites. Similar binding‐energy shifts have previously been associated with support‐induced electronic effects that may contribute to improved HER activity [21, 22, 36, 37].

**FIGURE 4:**
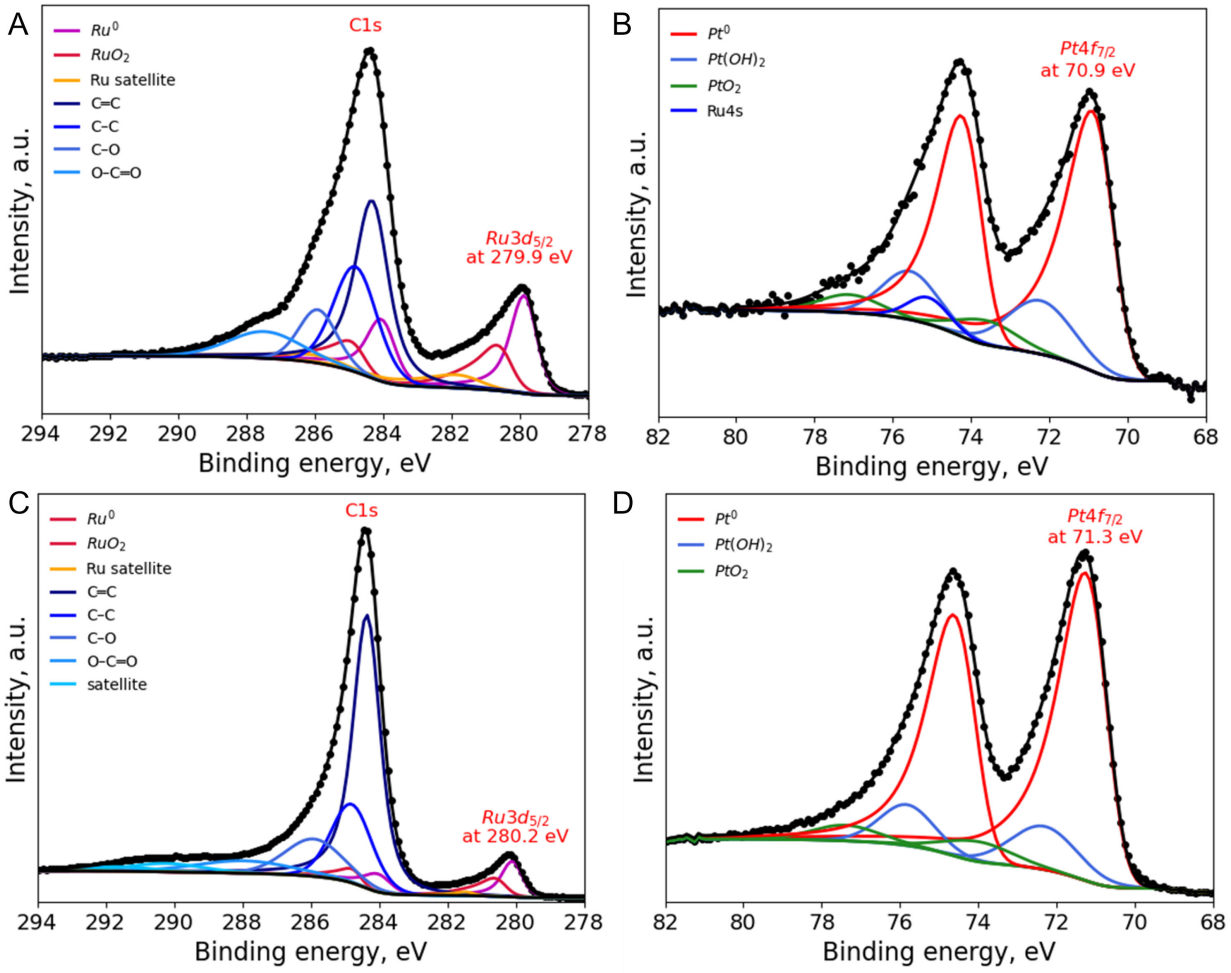
High‐resolution XPS spectra and peak fitting of the C 1s/Ru3d (A,C) and Pt 4f (B,D) regions for Ru–Pt/TiON–C (A,B), Ru/C (C), and Pt/C (D) samples.

Further insight can be obtained by comparison with our previously studied monometallic TiON‐supported systems. For Ru/TiON─C, a negative shift of 0.25 eV in the Ru° 3d5/2 binding energy relative to Ru/C was previously observed [22], comparable to the 0.3 eV shift found for Ru in the present Ru─Pt/TiON─C catalyst. This similarity suggests that the Ru─TiON interaction provides a major contribution to the modified electronic environment of Ru. For Pt/TiON─C, the Pt° 4f7/2 component previously exhibited a negative shift of 0.2 eV relative to Pt/C [21], whereas a more pronounced shift of 0.4 eV is observed for Pt in Ru─Pt/TiON─C relative to Pt/C in the present study. The larger relative shift in the bimetallic system is consistent with an additional modification of the Pt electronic environment associated with the presence of Ru. Overall, these trends indicate that the electronic structure of Ru─‍Pt/TiON─C is influenced by the combined contributions of metal–support interactions and bimetallic Ru─Pt interactions, although the relative contribution of each effect cannot be quantitatively resolved from the present XPS data.

### 3.2 Electrocatalytic HER Investigations

Electrocatalytic properties of the Ru─Pt/TiON─C sample were investigated in an acidic electrolyte (0.1 M HClO_4_) and systematically compared with a series of benchmark catalysts (Figure 5). Compared to the carbon‐supported Ru/C and Pt/C benchmarks, the Ru─Pt/TiON─C composite shows higher HER activity, indicating the benefits of a catalyst structure involving TiON support and Ru─Pt active sites. Furthermore, monometallic Ru/TiON─C and Pt/TiON─C composites with equal single metal loadings (i.e., 6 wt.% Ru and 2 wt.% Pt) were also included in comparison. Polarization curves given in Figure 5a show that Ru/TiON‐C is more active than Ru/C, underscoring the effect of the TiON support. At the same time, Ru/TiON‐C is significantly less active than Ru─Pt/TiON─C, indicating the benefits of the addition of minor amounts of Pt. Pt/TiON─C (with 2 wt.% Pt) shows a much lower catalytic effect, meaning that low‐loaded Pt active sites on TiON─C support cannot provide the observed HER activity of Ru─Pt/TiON─C by themselves. The overpotential needed to reach a current density of 10 mA cm^−2^ (*η*_10_) is often used to compare the HER performance of electrocatalysts. Ru─Pt/TiON─C is featured with the *η*_10_ value of only 11 mV, slightly lower than that of the Pt/C reference (14 mV). The mass activity comparison further demonstrates the feasibility of the Ru─Pt/TiON─C catalyst, Figure 5b, which exhibits much higher MA compared to the Pt/C reference due to the presence of ~11‐fold lower Pt loading and 2.7 times lower total metal loading. Such efficient metal utilization is central for potential industrial applications, considering that Pt and Ru are listed among critical raw materials. Furthermore, the turnover frequencies (TOFs) were evaluated to compare the intrinsic HER activities of the investigated catalysts. The TOF values were calculated assuming that the total metal content in the catalysts contributes to the reaction according to

**FIGURE 5:**
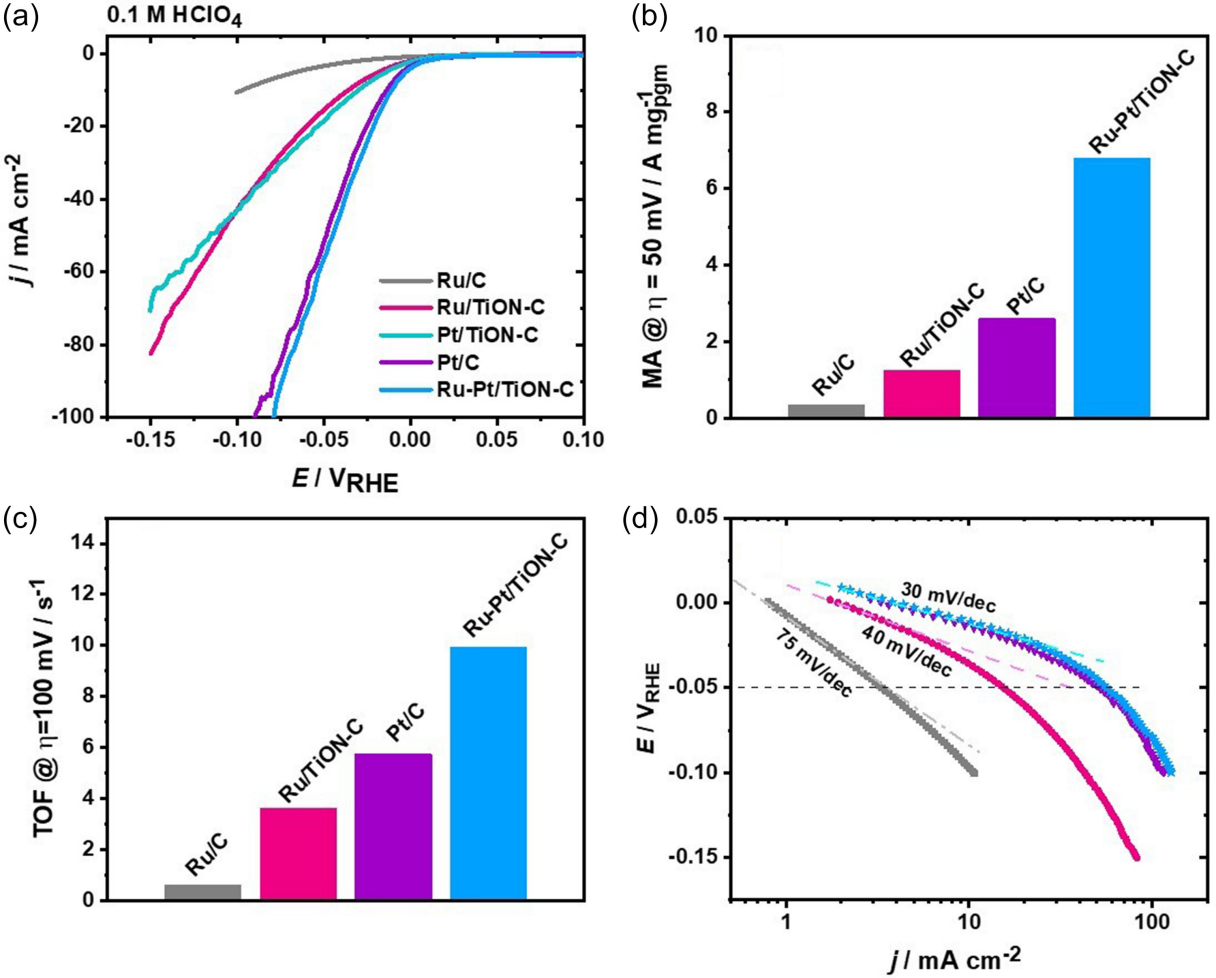
Comparison of the HER activities in acidic electrolyte: (a) polarization curves (Ar‐saturated 0.1 M HClO_4_, 10 mV/s, 1600 rpm); (b) corresponding mass activities; (c) turnover frequencies; and (d) Tafel slope analysis.

$$\mathrm{TOF}=\;\frac{i}{2Fn}$$

where *i* is the measured HER current, *F* is the Faraday constant (96,485 C mol^−1^), and *n* is the number of moles of active sites.

With this approach, the obtained TOFs represent apparent, lower‐bound values likely underestimating the true intrinsic activity since only a fraction of the metal atoms is expected to be electrochemically accessible active sites. Nevertheless, because the precise number and nature of active sites cannot be unambiguously determined for such bimetallic catalysts, this normalization provides a consistent and meaningful basis for comparing the intrinsic activities of the investigated materials. As shown in Figure 5c, the Ru─Pt/TiON─C composite exhibits the highest intrinsic activity among all studied catalysts, surpassing the Pt/C benchmark. Tafel analysis was performed to gain insights into HER kinetics (Figure 5d). Ru/C exhibits a Tafel slope of 80 mV/dec, indicative of sluggish reaction kinetics, which can be associated with strong H‐intermediate binding on Ru, often discussed in terms of the Gibbs free energy of hydrogen adsorption (Δ*G*_H*_). Interestingly, Ru/TiON─C shows a reduced Tafel slope of 40 mV/dec, suggesting a beneficial effect of the TiON support on the Ru active sites and faster reaction kinetics consistent with a Volmer–Heyrovsky pathway. Ru─Pt/TiON─C exhibits a Tafel slope of 30 mV/dec, identical to that of Pt/C, suggesting rapid reaction kinetics consistent with a Volmer–Tafel mechanism. In contrast, Pt/TiON─C exhibits a Tafel slope of 40 mV/dec, which can be attributed to the reduced Pt site density and the altered local environment of Pt at low loading, leading to a different reaction mechanism. Overall, these results indicate that TiON‐supported low‐loaded Pt sites alone are not sufficient to provide the same HER performance as observed for Ru─Pt/TiON─C, which is provided by the synergistic interaction between TiON, Ru, and Pt. A summarized comparison of the main HER performance descriptors is given in Table S1, suggesting that the Ru─Pt/TiON─C composite is a very promising catalyst for HER in PEM water electrolysis.

The stability of electrocatalytic materials is essential for their potential practical applications. In electrolyzers, both potentiodynamic and galvanostatic conditions can appear and provoke degradation of the catalysts, and thus, both types of degradation tests were applied to the Ru─Pt/TiON─C catalyst (Figure 6). After extensive potential cycling (2500 cycles) in the HER region, no activity decay was observed (Figure 6a). Similarly, during the 24 h constant‐current test (Figure 6b), the potential exhibited a gradual shift toward more negative values, without abrupt changes or sudden performance loss, indicating sustained HER operation throughout the investigated period. Part of the observed potential drift may also originate from the gradual accumulation and entrapment of hydrogen bubbles within the porous catalyst layer, which can decrease the electrochemically accessible surface area [38] and increase local resistance [39] during continuous HER operation. It is well established that degradation of supported PGM nanoparticles during HER can involve particle migration, agglomeration, and detachment from the support [40]. Accordingly, postmortem electron microscopy provides direct insight into possible structural degradation pathways.

**FIGURE 6:**
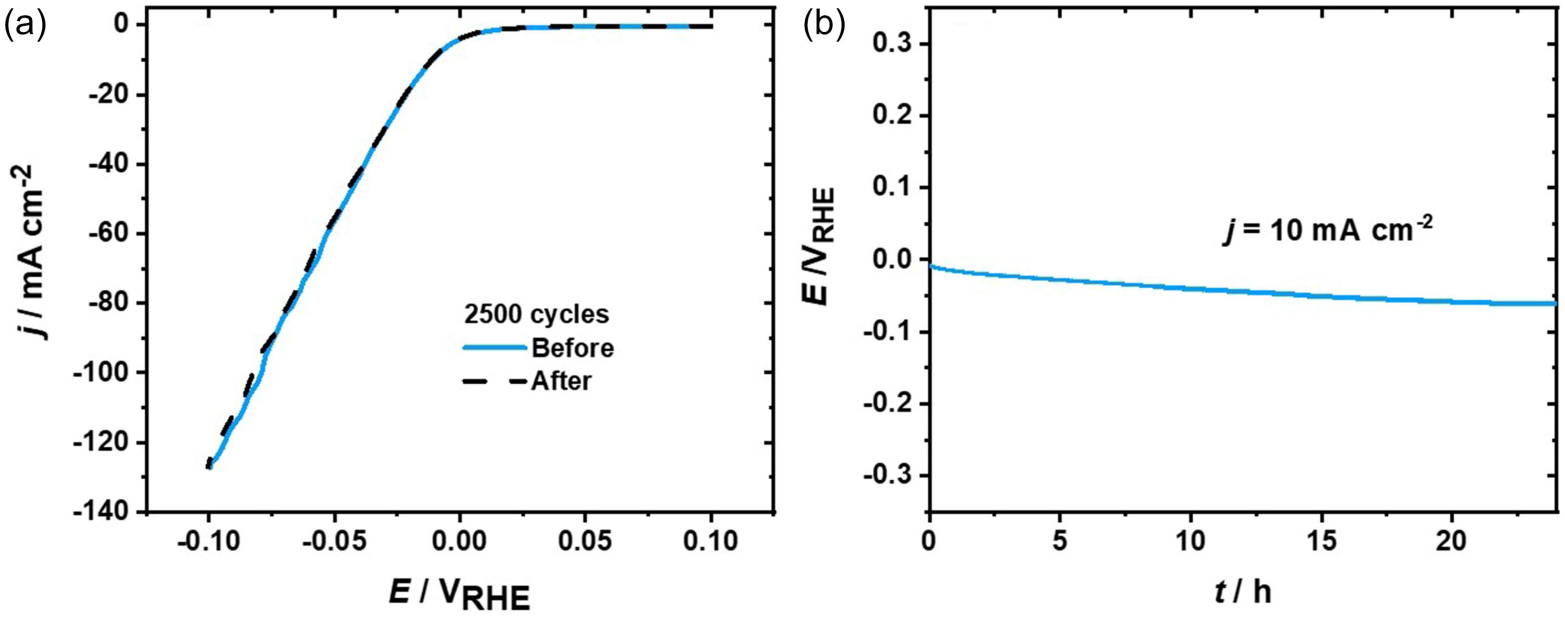
Stability tests for Ru─Pt/TiON─C in 0.1 M HClO_4_: (a) HER polarization curves recorded at 10 mV s^−1^ before and after 2500 potential cycles between −0.1 and 0.1 *V*_RHE_ at a scan rate of 100 mV s^−1^; (b) galvanostatic stability test at *j* = 10 mA cm^−2^.

The structural stability of the catalyst following prolonged HER operation in acid media was analyzed postmortem by SEM and STEM in combination with EDXS. Postmortem SEM‐EDXS analysis showed no pronounced large‐scale agglomeration or segregation of the metallic components, while Ru and Pt remained broadly distributed throughout the investigated catalyst layer (Figure S3). To evaluate the structural stability at the nanoscale, STEM imaging was additionally performed on the catalyst recovered after the 24 h constant‐current durability test. No pronounced nanoparticle agglomeration or substantially increased particle size was observed (Figure S4). Detailed STEM analysis coupled with EDXS elemental mapping (Figure 7) showed that the characteristic Ru─Pt particle architecture was largely preserved after prolonged HER operation. In particular, the spatial distribution of Ru and Pt and their intimate contact with TiON support remained comparable to those observed in the pristine catalyst (Figures 2 and 3).

**FIGURE 7:**
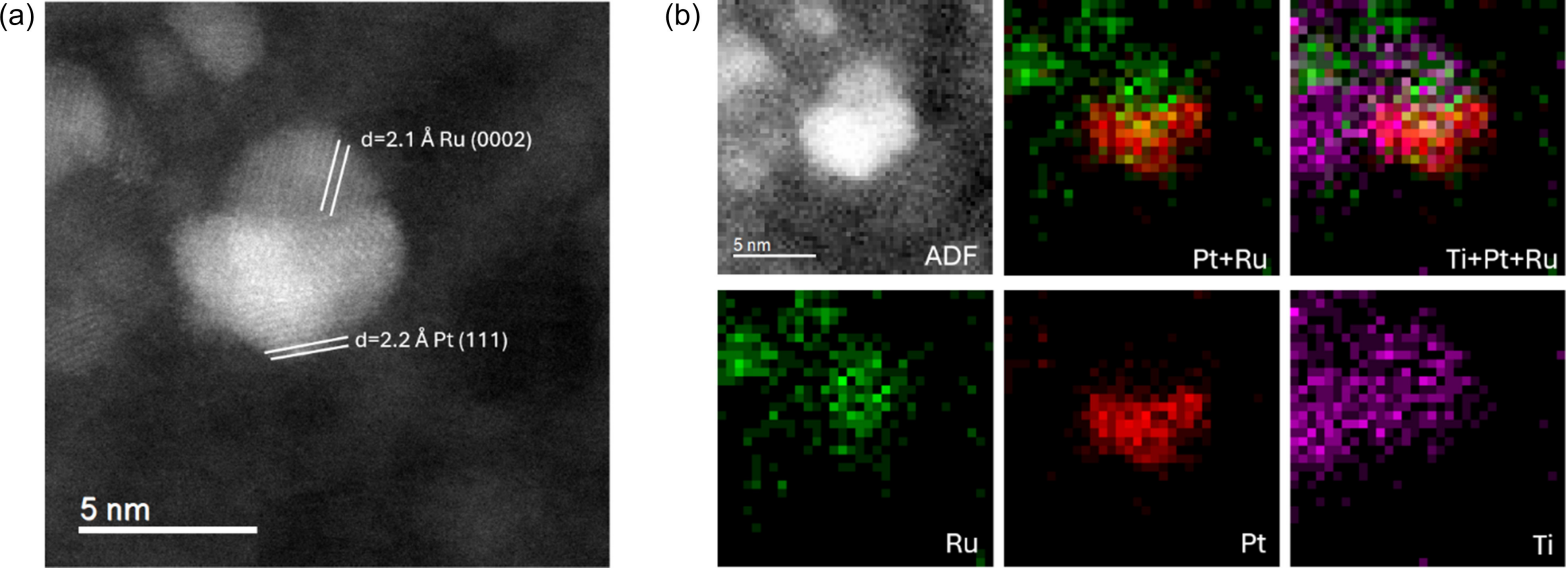
(a) ADF imaging of a Ru─Pt nanoparticle after a 24 h constant‐current durability test. The nanoparticle retains the Janus dumbbell‐type characteristics. (b) ADF imaging and corresponding EDXS elemental maps for Ru, Pt, and Ti.

Further, the Ru─Pt/TiON─C composite was subjected to similar HER performance tests in an alkaline electrolyte (Figure 8), while corresponding HER activity descriptors are summarized in Table S1. Comparison of HER polarization curves (Figure 8a) reveals that Ru─Pt/TiON─C is the most active catalyst for HER, followed by Ru/TiON─C and Pt/C. Low‐loaded Pt/TiON─C delivers low HER activity in alkaline media, again confirming the necessity of the synergistic effect of all three components in the Ru─Pt/TiON─C composite. In terms of mass activity, Ru─Pt/TiON─C and Ru/TiON─C show very similar performance (Figure 8b), as well as in the case of TOF comparison (Figure 8c). In terms of Tafel analysis (Figure 8d), Ru─Pt/TiON─C shows a slope of 30 mV/dec, corresponding to the Tafel slope in acid media and the Volmer‐Tafel route (Figure 5d). At the same time, Pt/C and Ru/TiON─C are featured with a slope of 40 mV/dec, indicating the Volmer–Heyrovsky reaction pathway. It can be proposed that Ru‐containing sites contribute to water activation, whereas adjacent Pt sites participate in hydrogen adsorption and evolution, consistent with literature reports on bifunctional Ru─Pt catalysts [24, 25, 26]. Comparison of HER activities of Pt/C and Ru─Pt/TiON─C in acid and alkaline media is shown in Figure S5. The activity of Pt/C is significantly lower in alkaline media than in acid (Figure S5a) due to the kinetic penalty caused by the sluggish water dissociation. For Ru─Pt/TiON─C, although still existing, this kinetic penalty decreases compared to the Pt/C reference (Figure S5b).

**FIGURE 8:**
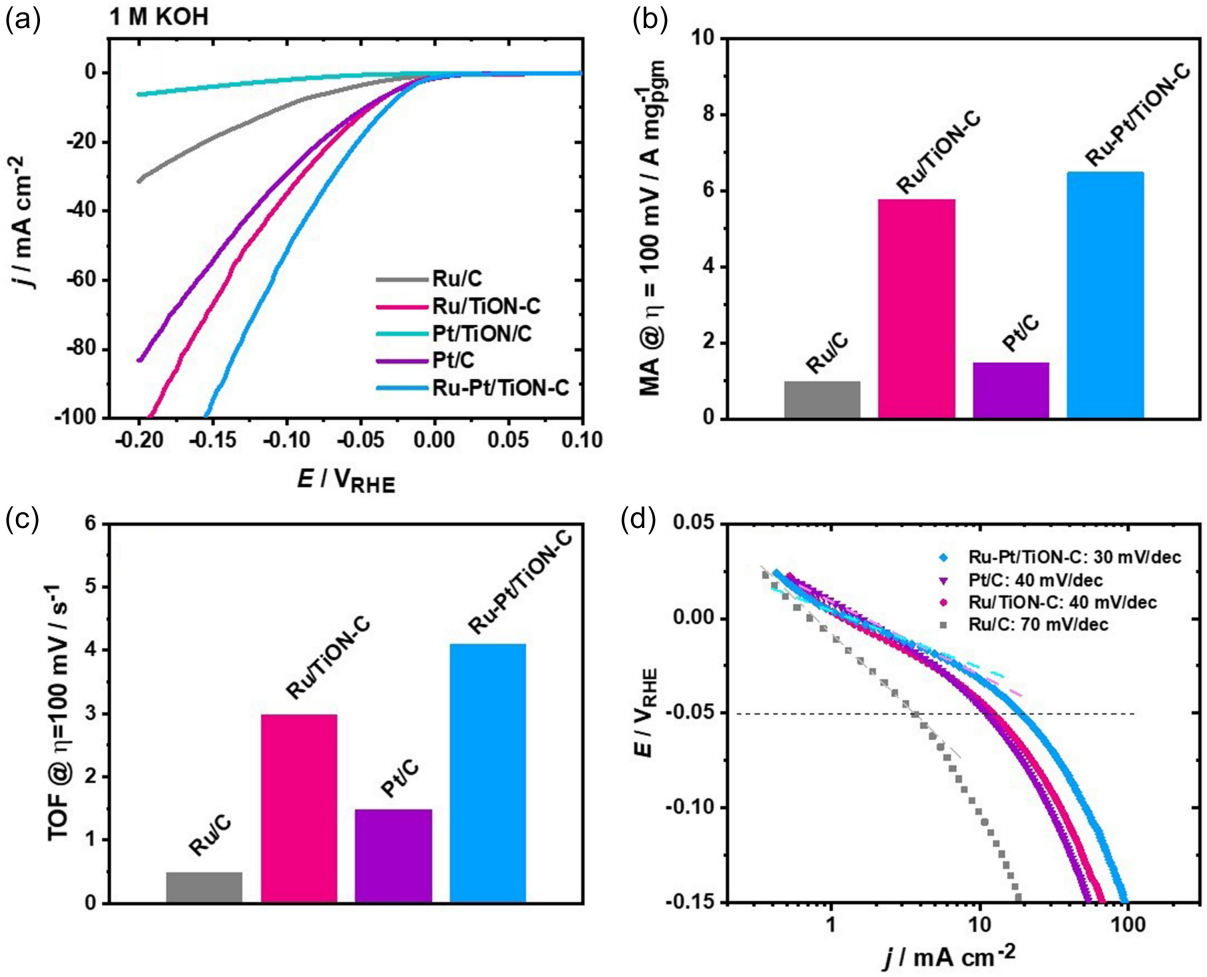
Comparison of the HER activities in alkaline electrolyte: (a) polarization curves (Ar‐saturated 1 M KOH, 10 mV/s, 1600 rpm); (b) corresponding mass activities; (c) turnover frequencies; and (d) Tafel slope analysis.

The stability of the Ru─Pt/TiON─C catalyst in alkaline media was evaluated using the potentiodynamic degradation test, Figure S6. Since the evolved hydrogen bubble adherence was strongly pronounced in alkaline media, the catalyst loading was reduced by 50% to decrease film thickness and facilitate bubble detachment. Even in that case, the attempted galvanostatic test was significantly affected by the physical accumulation of the hydrogen bubbles at the electrode surface (even under high rotation rates), leading to a rapid overpotential increase and fluctuations. However, the potentiodynamic degradation test was less susceptible to this issue, most likely due to the excursion to the non‐HER potential region (i.e., between 0.1 and 0 *V*_RHE_) during potential cycling, allowing time for accumulated bubbles to detach from the electrode surface. The results shown in Figure S6 demonstrate good alkaline durability of the Ru─Pt/TiON─C catalyst under the employed experimental conditions.

The results presented in Figures 5 and 8 (and the summary in Table S1) indicate that the Ru─Pt/TiON─C catalyst outperforms the Pt/C benchmark in both acid and alkaline media. Comparison with literature data for similar bimetallic Ru─Pt catalysts is reported in Table 1. In terms of *η*_10_ and Tafel slope parameters, the Ru─Pt/TiON─C catalyst compares fairly well with the literature data, although some exceptional values have been reported [25, 42, 45]. However, *η*_10_ values can be significantly impacted by the loading of the catalyst on the electrode, as discussed in more detail in our previous work [22]. Therefore, comparison of mass activities and TOFs provides a fair comparison of metal utilization and intrinsic catalytic properties of the active sites. In terms of these two parameters, the Ru─Pt/TiON─C is among the best‐performing Ru─Pt catalysts in both acid and alkaline media, although it is worth noting that many of these works do not report MA or TOF.

**TABLE 1:** Comparison of the HER performance of Ru─Pt/TiON─C with literature data.

Parameter	*η*_10_ /mV	Tafel slope/mV dec^−1^	MA/A mg^−1^	TOF/s^−1^
Catalyst				
**Acid electrolytes**				
**This work**	**11**	**30**	**17.0** (*η* = 100 mV)	**9.93** *(* *η* = 100 mV)
Ru_*x*_Pt_*y*_@rGO [25]	6	16.5	17.2 (*η* = 100 mV)	NA
Pt_1_Ru_5_@NH_2_‐rGO [26]	54/3^a^	65/46^a^	NA	NA
Ru@C/Pt_0.5_ [41]	32	26	NA	5.6 (*η* = 32 mV)
PtRu/mCNT [42]	17	54	NA	NA
PtRu/NCNT [43]	17	54	~0.7 (*η* = 50 mV)^b^	NA
**Alkaline electrolytes**				
**This work**	**32**	**30**	**6.47** (*η* = 100 mV)	**4.1** (*η* = 100 mV)
Pt/Ru nanowires [24]	24	26.3	0.289 (*η* = 50 mV)	1.42 (*η* = 50 mV)
Ru_*x*_Pt_*y*_@rGO [25]	9	20.9	5.82 *(* *η* *=* 100 mV)	NA
Pt_*x*_Ru_*y*_@NH_2_‐rGO [26]	310	169	NA	NA
WO_*X*_/RuPt/C [44]	19	34	3.78 (*η* =50 mV)	13.8 (*η* = 50 mV)
RuPt/C‐200 [45]	3.8	16.6	3.6 (*η* = 60 mV)	NA
PtRu/mCNT [42]	15	33.5	3.35 (*η* = 100 mV)	NA

a Before/after activation.

b Extrapolated by the authors of this work.

The enhanced catalytic performance of the Ru─Pt/TiON─C system is consistent with a synergistic effect between the Ru─Pt active sites and the TiON support. To gain further insight into the origins of HER activity, DFT calculations are used to evaluate hydrogen adsorption thermodynamics on the investigated catalyst systems.

### 3.3 DFT Calculations

A bimetallic Janus‐dumbbell Ru─Pt nanoparticle exhibiting Janus‐like characteristics was then created by combining the optimized monometallic clusters. Specifically, the relaxed Ru nanoparticle was first imported into VESTA, followed by the incorporation of the optimized Pt cluster using the phase editing tool. The Pt component was positioned relative to the Ru nanoparticle within a global Cartesian coordinate framework. This assembled Ru–Pt configuration served as the initial structure for further unconstrained geometry optimization in VASP, allowing complete relaxation of atomic positions. The final relaxed nanoparticle exhibited an approximate diameter of 1.9 nm as estimated from its spatial dimensions. This theoretically optimized nanoparticle size (~1.9 nm) is consistent with the experimental measurement of 1.95 nm, as confirmed experimentally by ADF imaging, as shown in Figure 1c).

A Ru─Pt nanoparticle consisting of 114 Ru atoms and 19 Pt atoms (average diameter >1.9 nm) was subsequently deposited on both TiON support and graphene support to model two catalyst–support interfaces. The resulting Ru:Pt atomic ratio (~6:1) is in close agreement with the experimentally reported composition based on metal loadings (5.5 wt% Ru and 1.85 wt% Pt), corresponding to an approximate ratio of ~5.7:1. The optimized configurations of the supported nanoparticles are presented in Figure 9.

**FIGURE 9:**
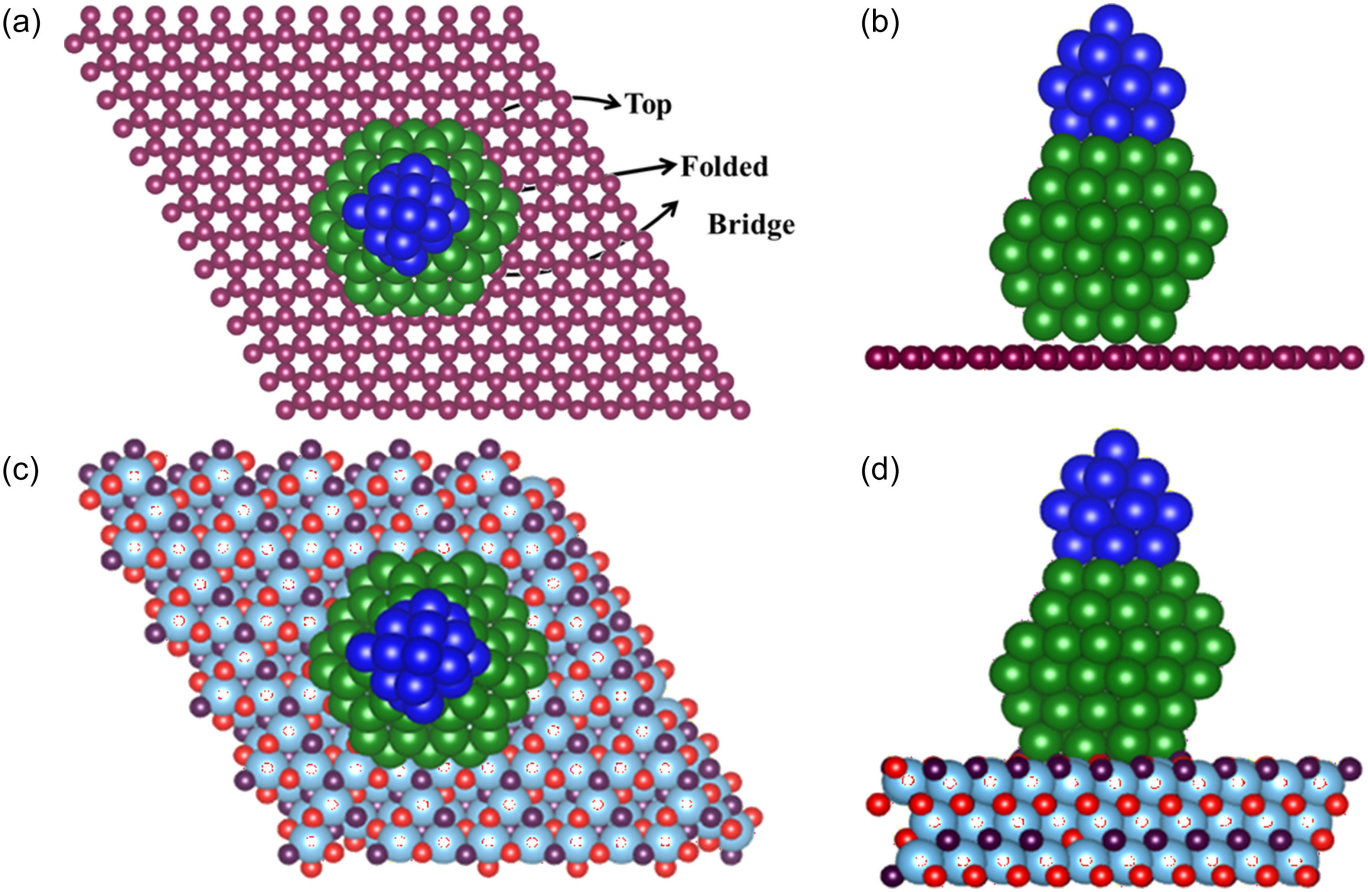
Optimized structures of Ru‐Pt‐graphene (a) top view (b) side views, and Ru‐Pt‐TiON (c) top view (d) side views.

To evaluate the influence of the support, which plays a key role in metal dispersion and catalytic activity, the interaction between the nanoparticle and the support was systematically investigated. Adsorption of the Ru─Pt nanoparticle on both graphene and TiON supports, as well as individual Pt adsorption on these supports, was examined. For all studied systems, the nanoparticle was oriented perpendicular to the support surface as this configuration demonstrated superior structural stability. Consequently, this orientation was adopted for all subsequent HER analyses.

The computational hydrogen electrode model [46] has been used to calculate the change in Gibbs free energy. In each step, free energy has been calculated by using the formula

$$\text{Δ}G=\;\text{Δ}E_{\mathrm{DFT}}+\;\text{Δ}E_{\mathrm{ZPE}}-T\text{Δ}S-\text{Δ}G_{\mathrm{pH}}-\text{Δ}H_{0K\to T}$$

Here, Δ*E*_DFT_ represents the total energy of the system calculated by DFT, $\text{Δ}E_{\mathrm{ZPE}}$ represents the zero‐point energy, TΔS represents the entropy corrections based on calculated vibrational frequencies of the system, and $\text{Δ}G_{\mathrm{pH}}$ represents the correction in Gibbs free energies due to pH, which is calculated by

$$\text{Δ}G_{\mathrm{pH}}=\ln10\;\times \mathrm{kBT}\times \mathrm{pH}$$

In this study, we assumed the pH value to be zero. Term $\text{Δ}H_{0K\to T}$ represents the change in internal energy due to the temperature, and its value is calculated by $\int_{0}^{T}C_{p}(T^{\prime })\;dT^{\prime }$, where $C_{p}(T^{\prime })$ represents specific heat at constant pressure, and the integral of this equation contributes negligibly at room temperature. The gas‐phase value was obtained from the thermodynamic tables found in textbooks [47].

The thermodynamic behavior of hydrogen adsorption on the Ru‍–‍Pt nanoparticle supported on TiON/C is illustrated in Figure 10. Figure 10a presents the adsorption thermodynamics at various active sites for the Ru─Pt/graphene system, while Figure 10b shows the corresponding results for the Ru─Pt/TiON system. It should be noted that the experimentally observed Ru─Pt nanoparticles (~2 nm) contain a large variety of distinct surface sites, including Pt‐rich, Ru‐rich, Ru─Pt interfacial, and metal–support interfacial environments. A comprehensive theoretical evaluation of all possible adsorption sites and their statistical distribution is beyond the scope of the present work. Therefore, the adsorption sites investigated in this study were selected as representative examples of Pt‐rich, Ru‐rich, and mixed coordination environments, allowing qualitative assessment of the effects of Ru incorporation and TiON support on hydrogen adsorption thermodynamics. The goal of the calculations is to identify general trends in adsorption behavior and provide atomistic insight into the experimentally observed HER performance rather than to establish the precise contribution of every individual surface site present in the catalyst. It should also be noted that the calculated Δ*G*_H*_ values correspond to isolated hydrogen adsorption under low‐coverage conditions. The present model does not explicitly account for coverage‐dependent adsorption effects, adsorbate–adsorbate interactions, mixed H/OH surface populations, or possible surface restructuring under HER operating conditions. Consequently, the reported adsorption energetics should be interpreted as qualitative thermodynamic descriptors intended to compare different catalyst configurations and identify general trends rather than provide a complete representation of the catalyst surface under realistic reaction conditions.

**FIGURE 10:**
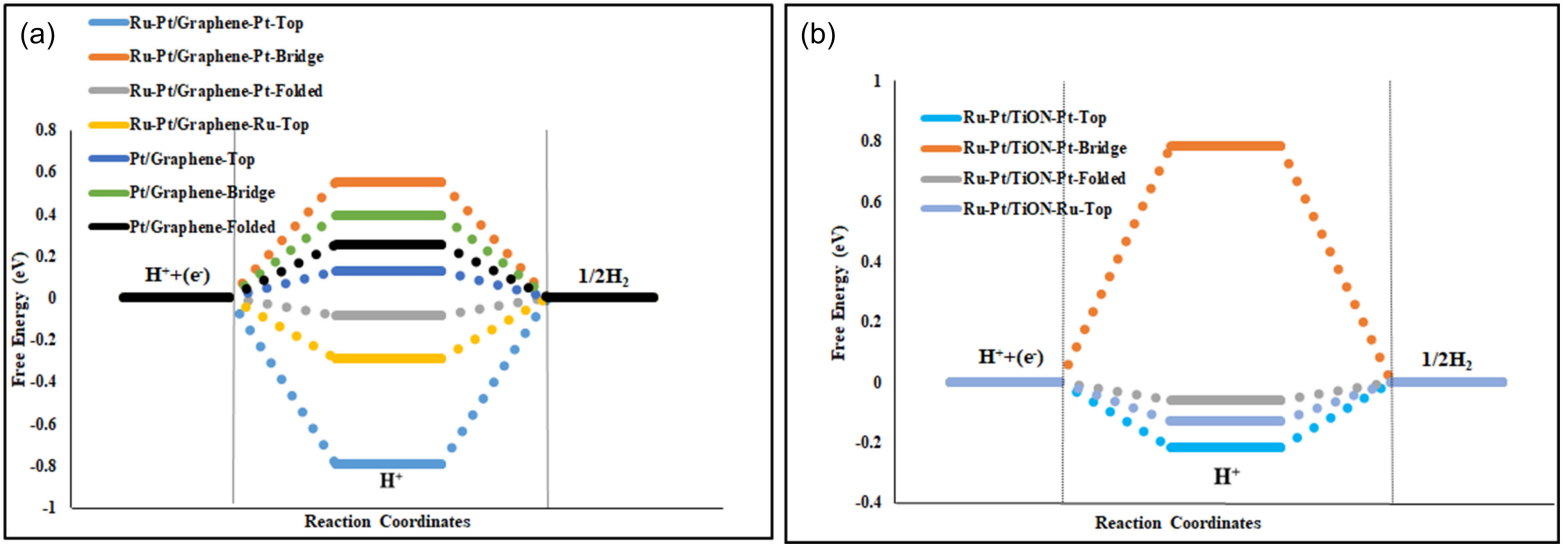
Thermodynamic behavior of hydrogen adsorption on the Ru─Pt nanoparticle supported on (a) graphene substrate and (b) TiON substrate.

The Pt/C system and Ru─Pt/C were evaluated by calculating the Gibbs free energy of adsorption (Δ*G*_H*_) at three representative adsorption active sites (Figure 10a): the top site on a Pt atom, the bridge site between two Pt atoms, and the folded site involving four Pt atoms within the nanoparticle. The corresponding Δ*G*_H*_ values were 0.29, 0.39, and 0.14 eV, respectively. Among these, the folded site exhibits the lowest Δ*G*_H*_ value, indicating relatively more favorable adsorption. However, since all values remain positive, hydrogen binding is weak across all sites, suggesting that the Pt–graphene system alone does not provide optimal thermodynamic conditions for HER.

To investigate the effect of bimetallic interactions, hydrogen adsorption was further analyzed on both Pt and Ru sites of a Pt─Ru nanoparticle supported on graphene in a perpendicular configuration. The calculated Δ*G*_H*_ values were −0.79 eV for the Pt site and −0.28 eV for the Ru site. These negative values indicate exergonic proton adsorption; however, the Pt site exhibits overly strong binding, whereas the Ru site shows comparatively moderate adsorption strength. To gain further insight, proton adsorption was also evaluated at different Pt sites within the same configuration. The Δ*G*_H*_ values for the top, bridge, and folded sites were −0.79, −0.55, and −0.08 eV, respectively. This indicates that the Ru─Pt/C composition exhibits more favorable hydrogen adsorption thermodynamics compared to the pure Pt/C composition, which is consistent with the experimentally observed HER trends. This result is aligned with the experimental activity trends observed in Figure 5a, where Ru─Pt/TiON─C exhibits higher HER activity than Pt/C.

To investigate the effect of bimetallic interactions coupled with the support effect, hydrogen adsorption was further analyzed on both Pt and Ru sites of a Pt─Ru nanoparticle supported on TiON in a perpendicular configuration (Figure 10b). The calculated Δ*G*_H*_ values were −0.21 eV for the Pt site and −0.13 eV for the Ru site. These negative values indicate exergonic proton adsorption; however, the Pt site exhibits overly strong binding, whereas the Ru site shows comparatively moderate adsorption strength. Proton adsorption was then evaluated at the top site of a Pt atom within the Ru─Pt/TiON configuration. The Δ*G*_H*_ values for the top, bridge, and folded sites were obtained to be −0.21, +0.78, and −0.058 eV, respectively. The bridge position offers endergonic proton adsorption, while the folded site offers the most thermoneutral adsorption.

The present DFT results are consistent with the experimental observations and suggest that the incorporation of Ru, combined with a favorable role of the TiON support (although explicit electronic‐structure analyses were beyond the scope of the present work), modifies hydrogen adsorption thermodynamics in the Pt─Ru/TiON system. It should be noted that the DFT investigations do not explicitly address additional descriptors relevant for alkaline HER (i.e., water adsorption, OH adsorption, and water dissociation energetics). Consequently, any discussion of the origins of high HER activity of Ru─Pt/TiON─C in alkaline media should be regarded as a mechanistic interpretation based on experimental observation and existing literature. Overall, the combined theoretical and experimental results suggest a beneficial interplay between bimetallic Ru─Pt active sites and the TiON support, highlighting the promise of the Ru─Pt/TiON─C system as a highly active low‐loading catalyst for HER across different electrolyte environments.

## 4 Conclusion

This work demonstrates that the combination of MSI and bimetallic active site engineering in a low‐loading Ru─Pt/TiON─C composite enables excellent HER performance in both acidic and alkaline electrolytes. Specifically, the Ru─Pt/TiON─C catalyst with only 1.85 wt.% Pt and 5.5 wt.% Ru achieves comparable activity to the 20 wt.% Pt/C benchmark in acid and superior performance in alkaline media while simultaneously exhibiting significantly enhanced metal utilization (mass activity) and intrinsic activity (turnover frequency). Comparison with Pt/TiON─C and Ru/TiON─C as control samples confirmed that a combination of bimetallic Ru─Pt active sites and MSI provided by TiON is necessary for effective HER performance. Characterization of Ru─Pt/TiON─C revealed that active sites are composed of bimetallic Janus‐dumbbell Ru─Pt nanoparticles with spatially segregated Ru‐ and Pt‐rich regions, whereas XPS analysis revealed shifts in the binding energies of Pt and Ru species, indicating a modified electronic environment consistent with support effects and Ru─Pt interactions. DFT calculations showed that incorporation of Ru modifies hydrogen adsorption thermodynamics in the Pt─Ru/TiON system, providing a possible explanation for the experimentally observed HER activity. In parallel, based on the previously established bifunctional effect in Ru─Pt systems, it can be proposed that Ru sites facilitate water dissociation, whereas neighboring Pt sites drive hydrogen adsorption, resulting in feasible HER in alkaline media. Our approach to designing highly efficient, low‐loaded PGM‐based catalysts can be expanded to a variety of materials and (electro)catalytic applications.

## Funding

This work was supported by the Javna Agencija za Raziskovalno Dejavnost RS (P2‐0393, I0‐0003, J2‐60043, J7‐70249, GC‐0004, N2‐0423, BI‐HR/25‐27‐013, BI‐BA/26‐27‐009), NATO Science for Peace and Security Program (G6230), Johannes Amos Comenius Program, European Structural and Investment Fund, Chemfells (No. CZ.02.01.01/00/22_010/0008809), Icelandic Infrastructure Fund (IREI), Icelandic research fund, and The Research Fund of the University of Iceland.

## Conflicts of Interest

The authors declare no conflicts of interest.

## Supporting information

Supplementary Material

## Data Availability

The data that support the findings of this study are available from the corresponding author upon reasonable request.
