# Supplementary material for: Janus Dumbbell Ru–Pt Nanoparticles on Titanium Oxynitride Boost Hydrogen Evolution Across pH
Source: ChemSusChem. 2026 Nov 8;19(21):e71203. doi: 10.1002/cssc.71203 (PMC13745868; doi:10.1002/cssc.71203)
Supplement: Supplementary file 1 [file CSSC-19-e71203-s001.pdf]

Supporting information

Mejrema Nuhanović[a,b], Ivan Marić[a,c], Marjan Bele[a*], Milena Šetka[d,e,f], Francisco Ruiz-Zepeda[a,d], Naveed Ashraf[g], Mohammad Isa[g], Younes Abghoui[g], Nejc Hodnik[a,b,d], Milutin Smiljanić[a*]

[a] *Department of Materials Chemistry, National Institute of Chemistry, Hajdrihova 19, 1000 Ljubljana, Slovenia*

[b] *University of Nova Gorica, Nova Gorica 5000, Slovenia*

[c] *Radiation Chemistry and Dosimetry Laboratory, Division of Materials Chemistry, Ruđer Bošković Institute, Bijenička 54,10000 Zagreb, Croatia*

[d] *Institute of Metals and Technology, Lepi pot 11, 1000 Ljubljana, Slovenia*

[e] *Device and Sensor Center, Fondazione Bruno Kessler, 38123 Trento, Italy*

[f] *Department of Inorganic Technology, University of Chemistry and Technology Prague, Technická 3, 166 28. Prague 6, Czech Republic*

[g] *Science Institute of the University of Iceland, Reykjavik, Iceland*

[*]Corresponding authors: milutin.smiljanic@ki.si; marjan.bele@ki.si

Characterization of the Ru-Pt/TiON-C sample

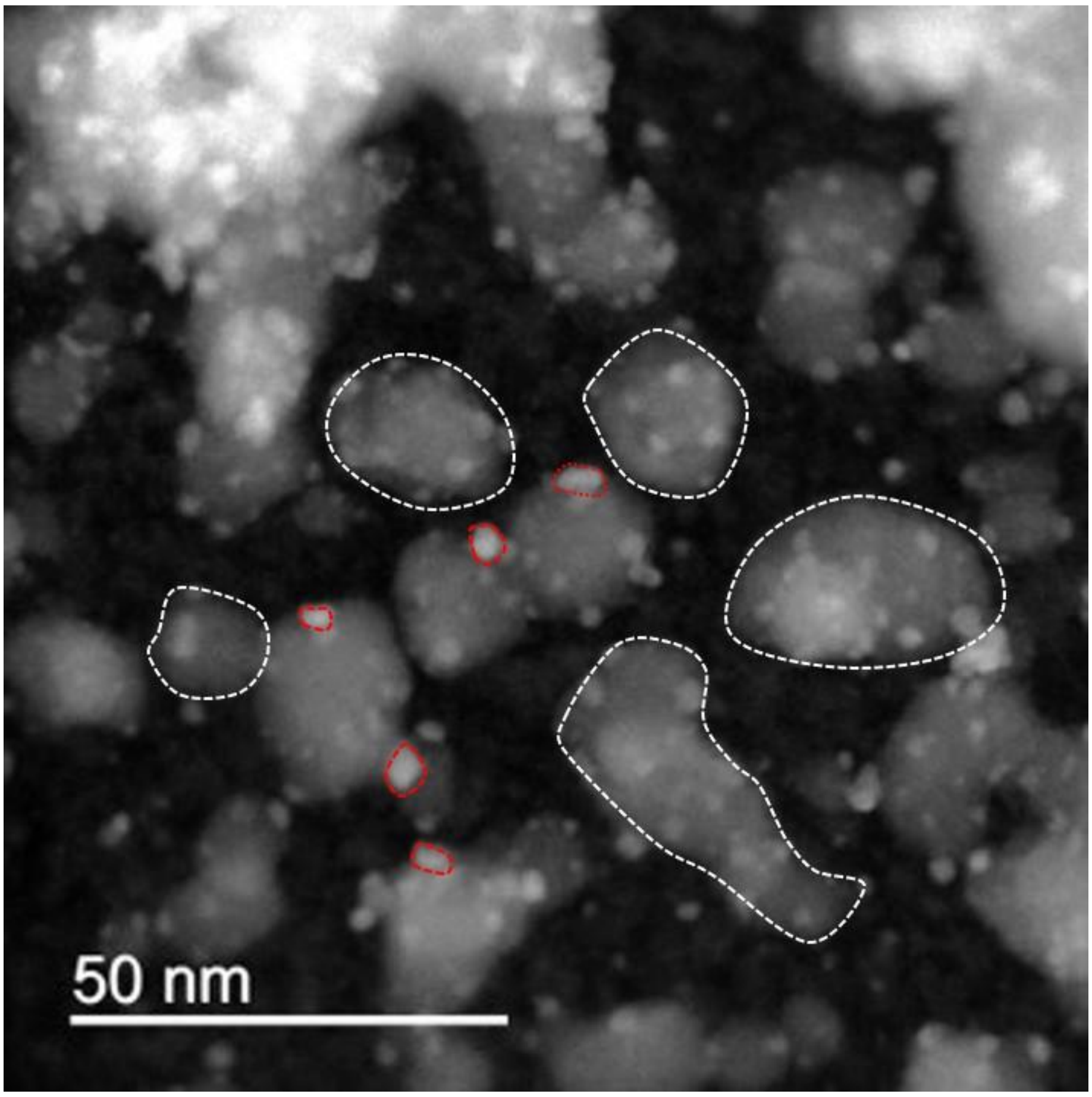

Figure S1. STEM imaging of Ru-Pt/TiON showing distinct TiON flakes (labeled with dotted white lines) and nanoparticles (labeled with red dotted lines).

Survey spectra for Ru-Pt/TiON-C, Ru/C, and Pt/C samples

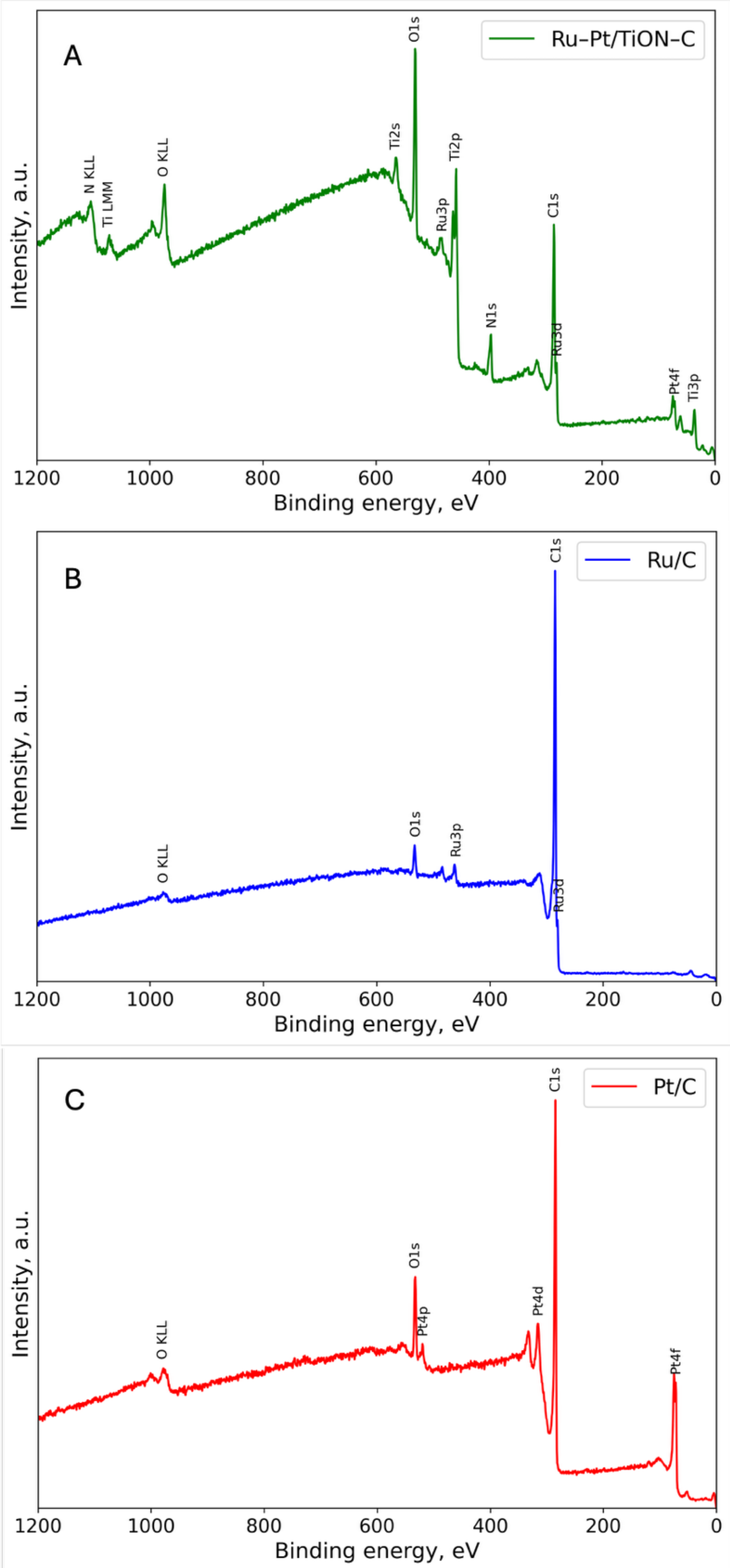

Figure S2. XPS survey spectra for: (a) Ru-Pt/TiON-C, (b) Ru/C and (c) Pt/C.

***SEM images and corresponding EDXS elemental maps of Ru–Pt/TiON-C after the 24 h HER durability test*.**

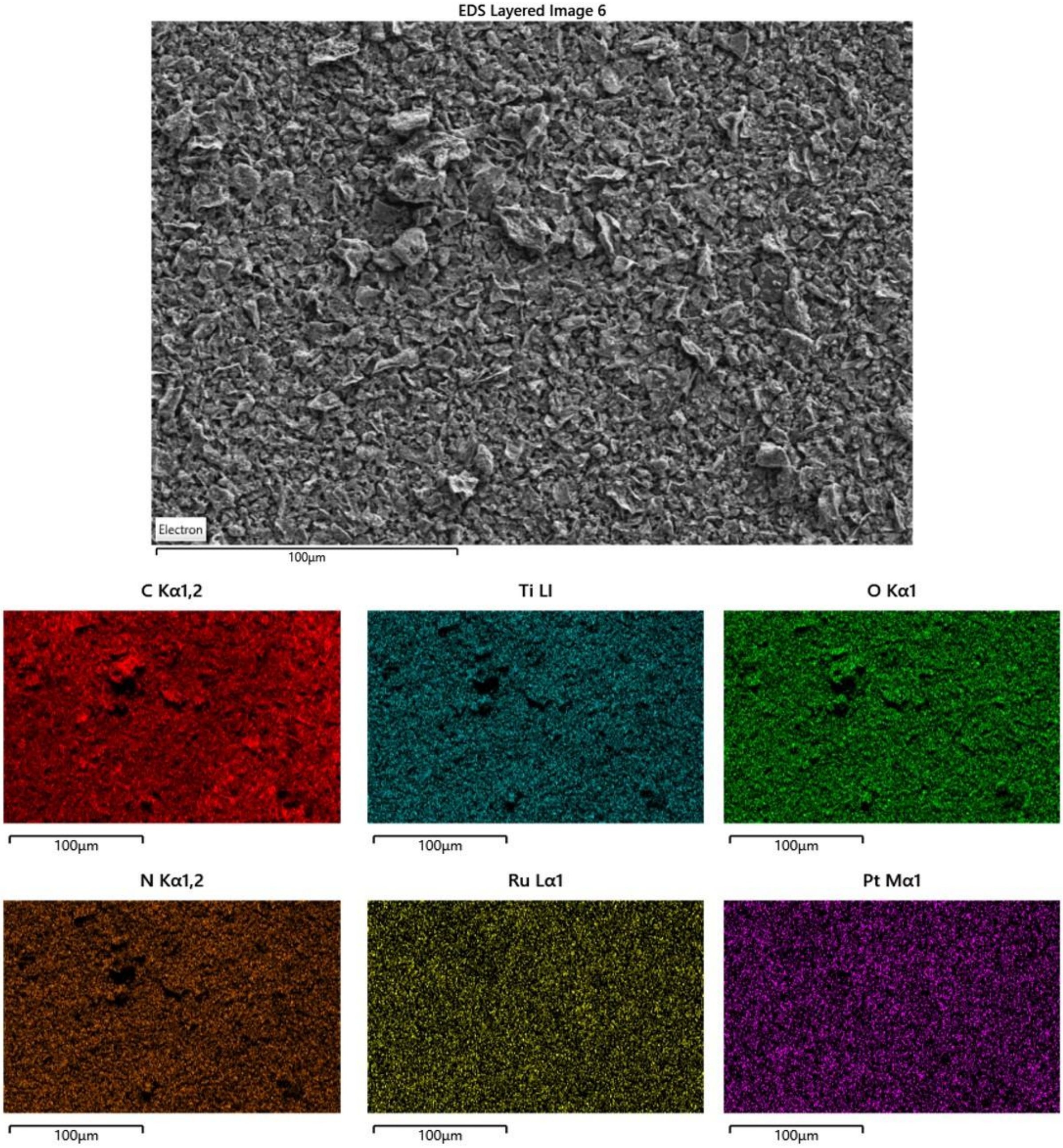

Figure S3. Post-mortem SEM image and corresponding EDXS elemental maps of Ru–Pt/TiON-C after the 24 h HER durability test.

Post-mortem STEM imaging of Ru–Pt/TiON-C after the 24 h HER durability test.

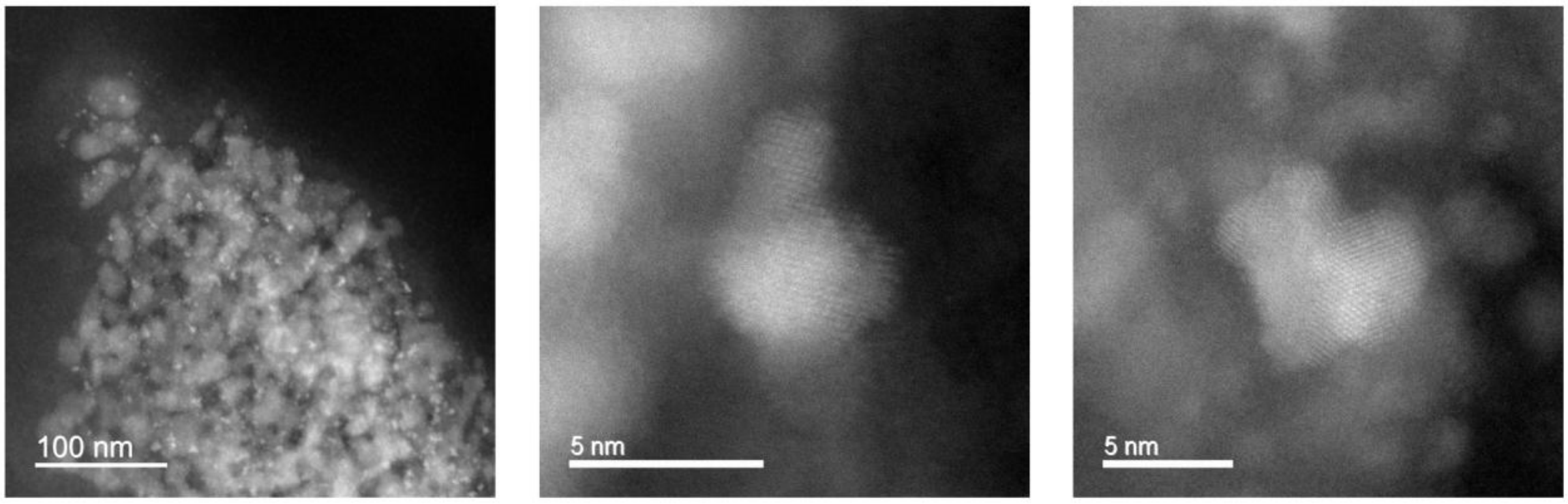

Figure S4. Post-mortem STEM ADF images at low and high magnification of Ru–Pt/TiON-C after the 24 HER durability test.

Comparison of the HER activity of Pt/C and Ru-Pt/TiON-C catalysts in acid and alkaline electrolytes

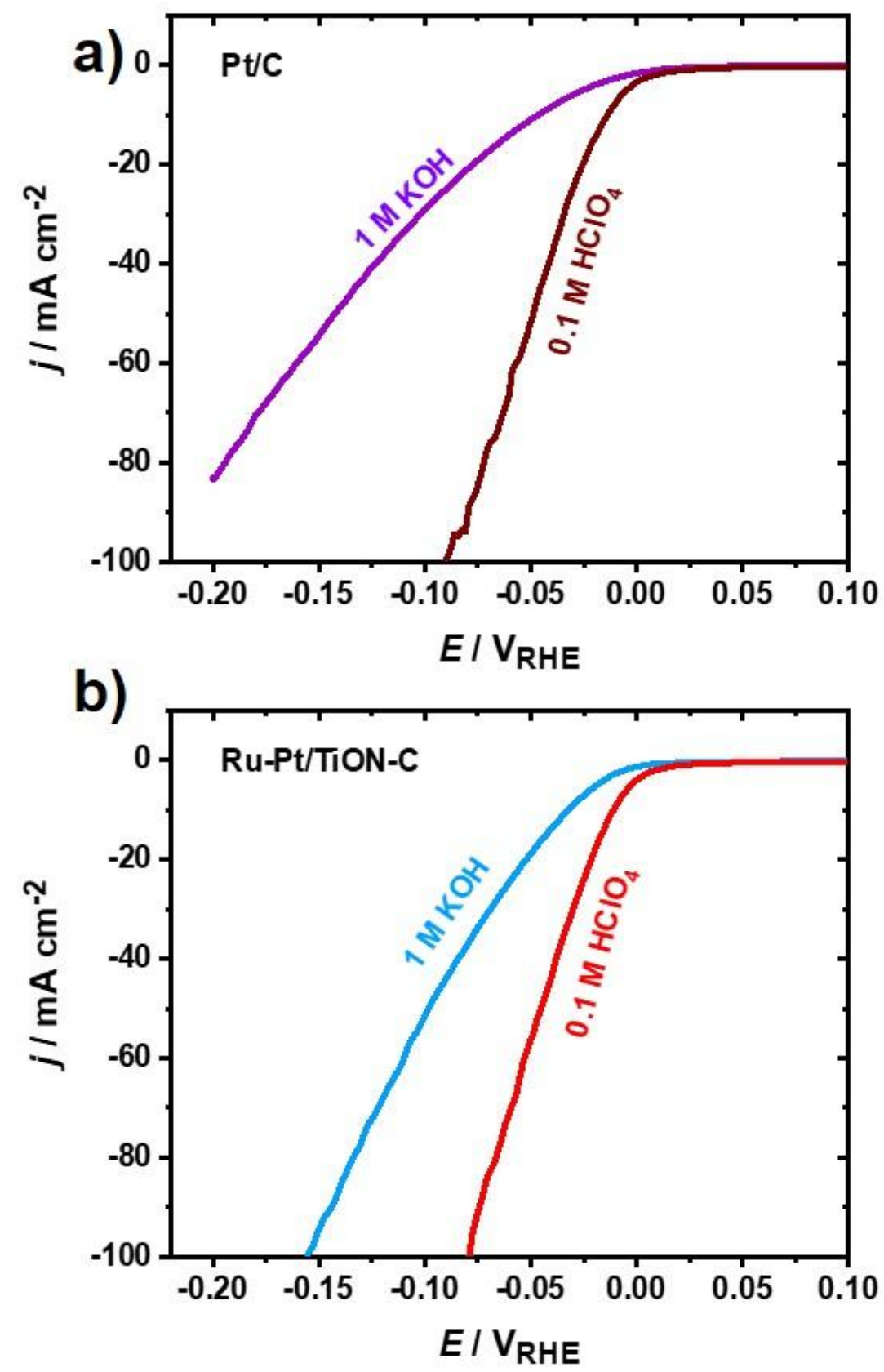

Figure S5. Comparison of HER polarization curves in 1 M KOH and 0.1 M HClO4 electrolytes (10 mV/s) for: (a) Pt/C and (b) Ru-Pt/TiON-C.

HER stability test in alkaline media

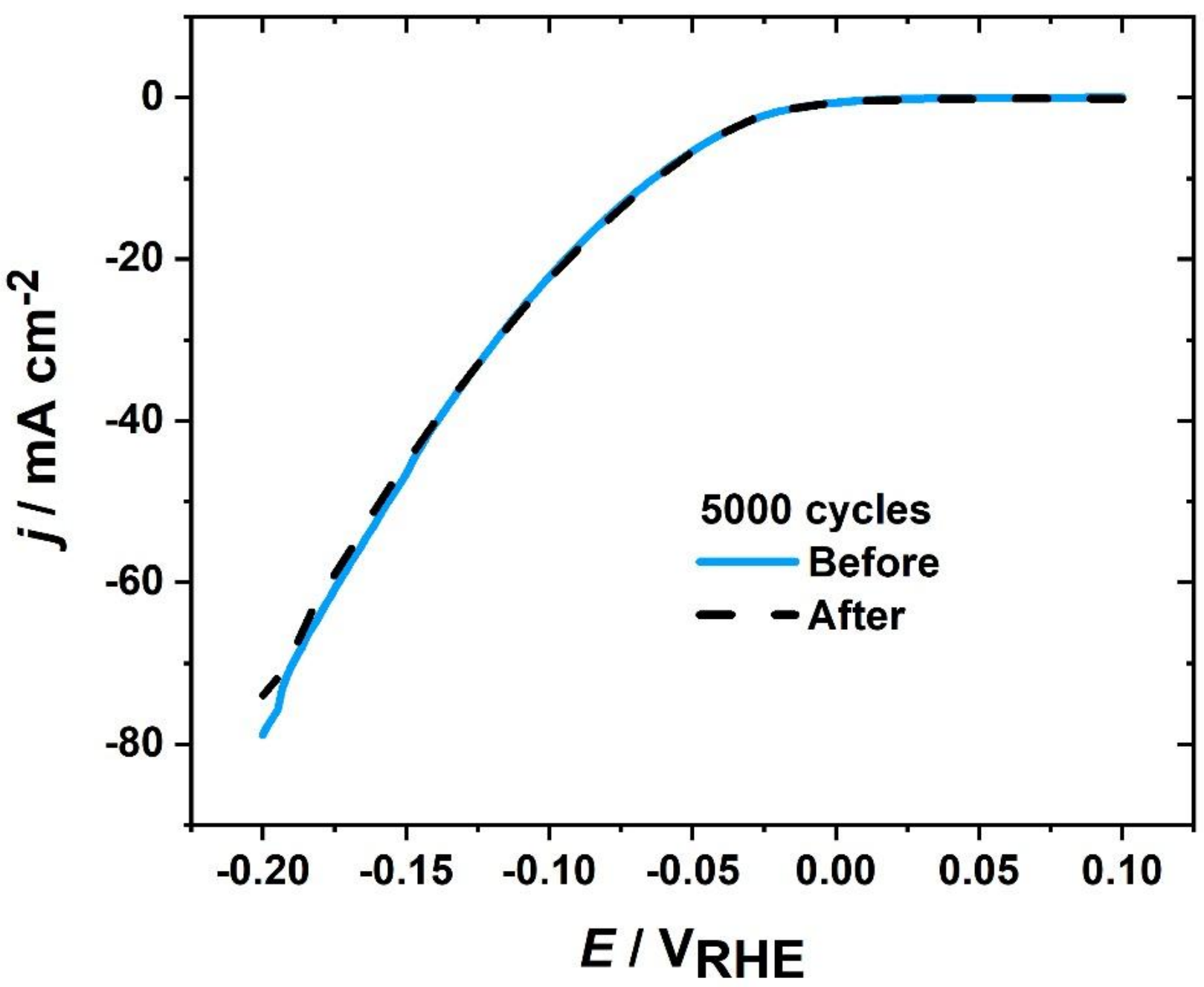

Figure S6. Stability test of Ru–Pt/TiON-C catalyst in 1 M KOH. Reported HER polarization curves were recorded at 10 mV s^{-1} before and after 5000 potential cycles between −0.2 and 0.1 V_{RHE} at a scan rate of 100 mV s^{-1}. The total catalyst loading was 51 µg cm^{-2}.

Comparison of the main HER activity descriptors for Ru/C, Pt/C, Pt/TiON-C, Ru/TiON-C, and Ru-Pt/TiON-C catalysts

Table S1. Comparison of the HER activity descriptors derived from Figures 5 and 7.

Catalysts / Parameter	Ru/C	Ru/TiON-C	Pt/TiON-C	Pt/C	**Ru-Pt/TiON-C**
**0.1 M $HClO_4$**					
η_{10} / mV	97	38	30	14	**11**
MA / A mg^{-1} @η=50 mV	0.33	1.23	NA	2.58	**6.8**
TOF / s^{-1} @η=100 mV	0.6	3.62	NA	5.73	**9.93**
Tafel slope / mV dec^{-1}	75	40	40	30	**30**
**1 M KOH**					
η_{10} / mV	103	42	NA	45	**32**
MA / A mg^{-1} @η=100 mV	0.97	5.77	NA	1.47	**6.47**
TOF / s^{-1} @η=100 mV	0.49	2.97	NA	1.48	**4.1**
Tafel slope / mV dec^{-1}	70	45	NA	45	**30**
